# Mechanisms of complement activation by dextran-coated superparamagnetic iron oxide (SPIO) nanoworms in mouse versus human serum

**DOI:** 10.1186/s12989-014-0064-2

**Published:** 2014-11-26

**Authors:** Nirmal K Banda, Gaurav Mehta, Ying Chao, Guankui Wang, Swetha Inturi, Liliane Fossati-Jimack, Marina Botto, LinPing Wu, Seyed Moein Moghimi, Dmitri Simberg

**Affiliations:** Division of Rheumatology, School of Medicine, University of Colorado Anschutz Medical Campus, 1775 Aurora Court, Aurora, CO 80045 USA; Moores UCSD Cancer Center, UC San Diego, 3855 Health Sciences Drive, La Jolla, CA 92093 USA; The Skaggs School of Pharmacy and Pharmaceutical Sciences, University of Colorado Anschutz Medical Campus, 12850 East Montview Blvd., Aurora, CO 80045 USA; Centre for Complement & Inflammation Research (CCIR), Division of Immunology and Inflammation, Department of Medicine, Imperial College London Hammersmith Campus, Du Cane Road, London, W12 ONN UK; Centre for Pharmaceutical Nanotechnology and Nanotoxicology, Department of Pharmacy, Faculty of Health and Medical Sciences, Universitetsparken 2, University of Copenhagen, DK-2100 Copenhagen, Denmark; NanoScience Centre, University of Copenhagen, DK-2100 Copenhagen, Denmark

## Abstract

**Background:**

The complement system is a key component of innate immunity implicated in the neutralization and clearance of invading pathogens. Dextran coated superparamagnetic iron oxide (SPIO) nanoparticle is a promising magnetic resonance imaging (MRI) contrast agent. However, dextran SPIO has been associated with significant number of complement-related side effects in patients and some agents have been discontinued from clinical use (e.g., Feridex™). In order to improve the safety of these materials, the mechanisms of complement activation by dextran-coated SPIO and the differences between mice and humans need to be fully understood.

**Methods:**

20 kDa dextran coated SPIO nanoworms (SPIO NW) were synthesized using Molday precipitation procedure. *In vitro* measurements of C3 deposition on SPIO NW using sera genetically deficient for various components of the classical pathway (CP), lectin pathway (LP) or alternative pathway (AP) components were used to study mechanisms of mouse complement activation. *In vitro* measurements of fluid phase markers of complement activation C4d and Bb and the terminal pathway marker SC5b-C9 in normal and genetically deficient sera were used to study the mechanisms of human complement activation. Mouse data were analyzed by non-paired t-test, human data were analyzed by ANOVA followed by multiple comparisons with Student-Newman-Keuls test.

**Results:**

In mouse sera, SPIO NW triggered the complement activation via the LP, whereas the AP contributes via the amplification loop. No involvement of the CP was observed. In human sera the LP together with the direct enhancement of the AP turnover was responsible for the complement activation. In two samples out of six healthy donors there was also a binding of anti-dextran antibodies and C1q, suggesting activation via the CP, but that did not affect the total level of C3 deposition on the particles.

**Conclusions:**

There were important differences and similarities in the complement activation by SPIO NW in mouse versus human sera. Understanding the mechanisms of immune recognition of nanoparticles in mouse and human systems has important preclinical and clinical implications and could help design more efficient and safe nano-formulations.

**Electronic supplementary material:**

The online version of this article (doi:10.1186/s12989-014-0064-2) contains supplementary material, which is available to authorized users.

## Introduction

Superparamagnetic iron oxide (SPIO) is one of the most widely cited metal oxide nanoparticle that has been used as magnetic resonance imaging (MRI) contrast agent alone and as a component of multifunctional nanomedicines [1]. Dextran SPIO consists of magnetite-maghemite (Fe_3_O_4_ and γ-Fe_2_O_3_) crystalline cores of 3–10 nm size coated with dextran or carboxymethyl dextran [2]. Despite the tremendous medical need in efficient MRI contrast agents [3], several dextran SPIO formulations have been withdrawn from the clinical use due to hypersensitivity in patients (Sinerem, Combidex, Feridex). Another problem of these nanomaterials is the propensity of dextran SPIO for liver and spleen clearance, which limits imaging to macrophage-rich organs. In order to design contrast agents with reduced toxicity and improved pharmacokinetics, a basic understanding of immune recognition of these materials in both mouse (preclinical) and human (clinical) systems of paramount importance.

The complement system accounts for about 5% of globulins in serum and is responsible for recognition, elimination and destruction of pathogens [4]. Activation of the complement on the foreign surface takes place via either the classical pathway (CP), the lectin pathway (LP) or the alternative pathway (AP). The CP activation is triggered via initial binding of IgG or IgM to the pathogen surface, followed by binding and activation of C1q component and formation of C4bC2a, a C3 convertase. C4bC2a cleaves C3 into C3a and C3b, and the latter covalently attaches via highly reactive thioester group to hydroxyls and amines on the foreign surface [5]. More C3b is formed through the alternative pathway (AP) via the formation of alternative C3 convertase C3bBb. Lectin pathway (LP) is somewhat different in mice vs. humans. In mice, the activation is primarily triggered via initial binding of mannose-binding lectin -A and -C or ficolin A to carbohydrates on the pathogen surface, leading to activation of MBL-associated serum protease MASP-2 and formation of C4bC2a, the C3 convertase. In humans, five different sugar recognition molecules have been identified that are able to initiate the LP: MBL, M-, L-, and H-ficolins; and collectin 11 (CL11 or CL-K1), but the downstream activation of the classical C3 convertase is believed to be similar in mice and humans [6].

Activation of the complement plays a major role in the immune recognition of nanoparticles and pathogens [7]. Opsonization by C3b and its cleavage products (e.g., iC3b) triggers recognition by complement receptors CR3 (also known as CD11b/CD18 or Mac-1), complement receptor CR4 (CD11c/CD18), and complement receptor immunoglobulin (CRIg) [8,9], leading to particle uptake by macrophages. Complement cleavage byproducts C3a and C5a are among the most potent anaphylatoxins and proinflammatory molecules with low nanomolar affinity [10]. Many nanoparticulate systems including iron oxides exhibit signs of the complement activation *in vivo* [11-20]. At the same time, despite the accumulating evidence on the involvement of complement in acute and often life threatening reactions observed in some patients infused with dextran SPIO, the mechanisms of complement activation are not clear. Our earlier report using shotgun proteomics demonstrated the absorption of the LP components MBL-A/C and MASP-1/2 from mouse plasma on the SPIO surface [21]. The involvement of the LP in the complement activation would be a logical assumption, since dextran is a polysaccharide and as such may be recognizable via the LP [22]. This contrasts the reported mechanisms of activation in human sera. Pedersen et al. demonstrated that iron oxide nanoparticles of large curvature activate the CP in human plasma [13] due to the presence of specific anti-dextran IgM antibodies in certain individuals. In view of the above-mentioned similarities and differences between mouse and human complement systems, we set out to systematically study the mechanisms and pathways of the complement activation in mouse versus human sera. For the study below we used our previously described 20 kDa dextran-coated SPIO nanoworms (SPIO NW) that have physicochemical and biological properties similar to Feridex [23,24]. Our data suggest that SPIO NW activate complement in mouse and human sera, but the mechanisms of activation are different, which could bear important implications on preclinical and clinical studies of these materials.

## Results and discussion

### Mechanisms of complement activation by SPIO in mouse sera

**Figure 1:**
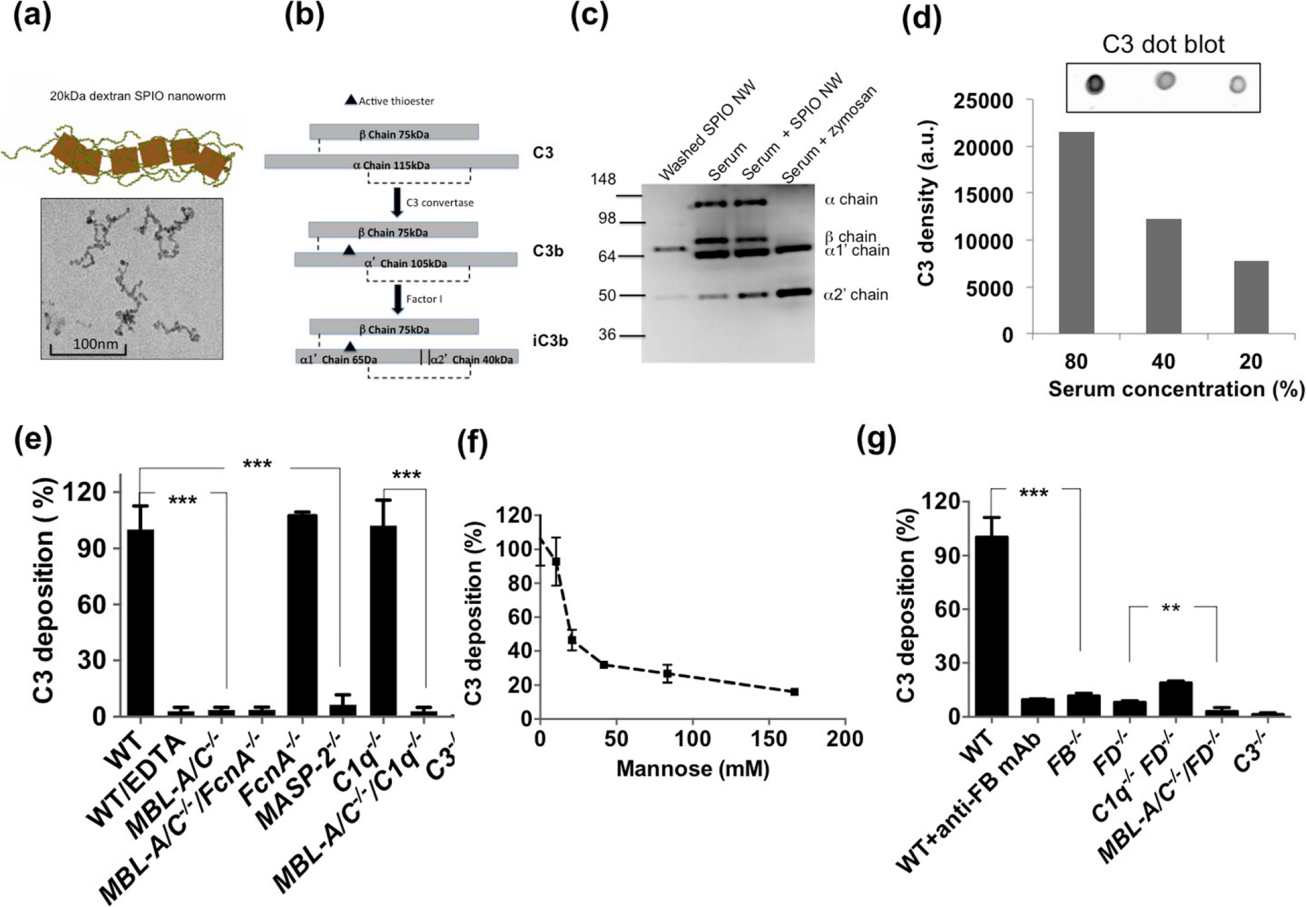
**SPIO-mediated complement activation in knockout mouse sera. (a)** Model of worm-like polycrystalline SPIO NW shows magnetite/maghemite crystals (10 nm, brown color) randomly coated with 20 kDa dextran chains (arrow). Size bar for transmission electron microscopy image: 100 nm; **(b)** scheme of C3 chains and cleaved fragments; **(c)** nanoparticles were incubated with normal mouse sera (C57BL/6) and C3 fragments were detected on purified SPIO NW or in whole serum by western blotting. Fragments of C3 are clearly detected in purified SPIO NW sample. Zymosan particles (1 mg/ml) also showed strong complement activation; **(d)** deposition of C3 fragments on SPIO NW surface as a function of normal serum concentration as detected with dot blot immunoassay (see Methods); **(e)** deposition of C3 fragments in sera deficient for the LP components shows complete dependency on the MBL-MASP-2 but not on FcnA. All experiments were repeated at least three times based on n = 3; **(f)** deposition of C3 fragments in normal mouse sera pre-incubated with different concentrations of the LP inhibitor mannose; **(g)** deposition of C3 fragments in sera deficient for the AP. N = 3 for each bar, repeated at least 3 times. ***p < 0.0001, and **p < 0.01.

Dextran SPIO contrast agents Feridex I.V.™ (Feridex) and Sinerem™ have been discontinued due to safety issues and are no longer available on the market. Although we used Feridex in our earlier studies [25], the remaining amount was not sufficient for a full scale complement study, and therefore we used our previously described dextran-coated SPIO nanoworms [23]. These nanoparticles are prepared by precipitation of 20 kDa dextran with FeCl_2_/FeCl_3_ using the established method of Molday and MacKenzie [26]. The same method was used for preparation of Feridex and other dextran SPIO [27,28], with the difference being that for Feridex manufacturing 10 kDa (T-10) dextran was used, whereas we used 20 kDa dextran. SPIO NW (Figure 1a and Additional file 1: Figure S1) have a worm-like shape with multiple crystalline cores (~6-7 nm each crystal) embedded in the dextran meshwork and with an average hydrodynamic diameter of 169 ± 77.43 nm and zeta potential of −6.05 ± 8.29 mV (Figure 1a). Our previous studies showed that Feridex and SPIO NW have similar physicochemical and immunological properties [24]. To verify the complement activation by SPIO NW and Feridex in mouse serum, we incubated the particles in normal mouse serum at the concentration similar to the concentrations used in vivo (100 μg/mL serum, or 4 mg/kg body weight), washed multiple times by ultracentrifugation and analyzed for the presence of complement fragments in serum supernatant and on the purified particles. The binding and activation of a complement leads to C3 cleavage and covalent deposition of C3b via active thioester bond on the pathogen surface. Main C3 fragments are schematically shown in Figure 1b. The C3 deposition and the pattern of C3 fragments on the surface of Feridex (Additional file 2: Figure S2) were similar to those of SPIO NW. Western blot analysis of fragments deposited on SPIO NW revealed presence of C3b α1’ and α2’ chains (Figure 1b lane 1), suggesting complement activation with subsequent cleavage of C3b to iC3b by Factor I [29]. Nanoparticle-treated serum showed an increased concentration of C3 fragments, compared to the non-treated sera (Figure 1c, lane 3). Zymosan (1 mg/mL) caused complete disappearance of α chain in serum, suggesting a much more potent activation of the complement than SPIO NW (Figure 1c, lane 4). The reason for the shifted position of α1’ chain eluted from SPIO NW could be due to binding of α1’ chain via thioester to high molecular weight components on the surface of SPIO NW (e.g., dextran). The reason for lack of detection of C3 β chain (~70 kDa) on washed SPIO NW and zymosan treated serum is not clear (albeit consistent), but could be due to a weaker immunoreactivity of the antibody toward the eluted β chain compared to the α chain fragments. Next, SPIO NW were incubated with different dilutions of sera (80%, 40%, 20%) and washed using ultracentrifugation. Dot blotting of washed SPIO NW on nitrocellulose membrane and immunodetection of C3 fragments showed linear decrease in C3 fragment deposition with decrease in serum concentrations (Figure 1d). There was no detectable C3 in the supernatant from the last wash of the nanoparticles, suggesting that dot blot assay is detecting the nanoparticle bound C3 fragments (shown in Figure 1c) and not the carryover protein. In the subsequent experiments, we used dot blot as the main method to quantify and compare the complement activation in mouse sera and will refer to all C3 fragments as “C3”.

In order to further investigate the pathways by which SPIO NW trigger mouse complement we examined C3 fragment deposition after incubation of SPIO NW with sera genetically lacking specific complement components. The serum was mixed with SPIO NW at a final iron concentration of 0.15 mg/ml and serum concentration of 75% v/v. There was a 97% decrease in C3 deposition in serum from wild type (WT) mice supplemented with 5 mM ethylenediamine tetraacetic acid (EDTA) (Figure 1e), since complement activation via all pathways requires both Ca^2+^ and Mg^2+^ ions. Also, there was not detectable C3 deposition in *C3*^*−/−*^ serum. Activation of the LP proceeds following the binding of serum MBL or the binding of serum Ficolin A (FcnA) to a LP-activating surface [30]. There was 95% less C3 deposition in *MBL-A/C*^*−/−*^ mouse serum compared to normal mouse serum (Figure 1e). There was a similar decrease in C3 deposition in the sera lacking both MBL-A/C and FcnA (*MBL-A/C*^*−/−*^*FcnA*^*−/−*^), and no decrease in *FcnA*^*−/−*^ serum (Figure 1e), confirming that complement activation in mouse serum depends on MBL-A/C and not on FcnA. Three different types of mannose-associated serine proteases (MASPs), i.e. MASP-1, MASP-2 and MASP-3 have been reported to be associated with MBL or ficolins in mouse sera [30,31]. In order to confirm the role of the LP in the complement activation, we measured C3 deposition using mouse serum deficient for MASP-2. In *MASP-2*^*−/−*^ mouse serum there was a significant (p <0.05) 91% reduction in C3 deposition on the surface of SPIO NW (Figure 1e). At the same time, there was no significant decrease in C3 deposition in the *C1q*^*−/−*^ serum (Figure 1e). C1q is required for the initiation of the CP of complement. There was nearly complete loss of C3 binding to SPIO NW in double knockout *MBL-A/C*^*−/−*^*C1q*^*−/−*^ serum, but no decrease of C3 binding in *C1q*^*−/−*^ serum compared to WT serum (Figure 1d), suggesting that the CP plays a minor, if any role, in mouse complement activation and C3 deposition. On the other hand, mannose, which is the inhibitor of the LP, decreased C3 deposition in a concentration-dependent fashion (Figure 1f). Combined, these experiments confirm the critical role of the LP in the complement activation on SPIO NW in mouse serum.

To determine the role of AP in the complement activation and C3 deposition, we used sera genetically deficient for critical components of the AP: factor D (FD) and factor B (FB). Following the initial deposition of C3b, the amplification via the AP takes place through the binding of FB to C3b, subsequent cleavage into Bb by FD and formation of C3bBb (alternative C3 convertase). According to Figure 1g, the deposition of C3 in *FB*^*−/−*^ serum as well as in serum in which FB was immunochemically depleted with a previously validated antibody [32,33] was 90% less than that of WT serum. C3 deposition was also decreased by 92% in the *FD*^*−/−*^ serum and by 82% in *C1q*^*−/−*^*FD*^*−/−*^serum. The higher deposition of C3 in the *C1q*^*−/−*^*FD*^*−/−*^serum compared to *FD*^*−/−*^ serum is interesting and could suggest compensatory activity of the LP in the double negative sera. C3 deposition in double negative *MBL-A/C*^*−/−*^*FD−/−*^*−*^ serum was decreased by 97% compared to WT serum and by 6% compared to *FD*^*−/−*^ serum. Collectively, these experiments confirm that in mouse sera the complement activation is initiated mainly via the LP and amplified via the AP, and that the amplification loop adds the majority of C3 deposited on the surface of SPIO. There is a possibility that MBL-A/C and/or MASP-1/2 could directly trigger the activation of the AP, as was suggested previously [34,35], but we did not investigate this hypothesis further. Some level of the complement activation that is not inhibited in *MBL A/C*^*−/−*^*FD*^*−/−*^ serum is probably due to a baseline spontaneous C3 hydrolysis and formation of C3H_2_O but we did not investigate this hypothesis further.

### Mechanism of complement activation by SPIO NW in human serum

Albeit mouse and human complement systems share similarities, the relative contribution of pathways to the complement activation could be different [36]. Therefore, we determined the contribution of each of the pathways using human serum. Since human sera deficient for the complement factors and components are not readily available, we used a previously established combination of depleted sera and purified complement factors [14]. In addition, due to the availability of commercial quantitative assays, we measured fluid phase markers rather than the C3 deposition for measuring complement activation.

**Figure 2:**
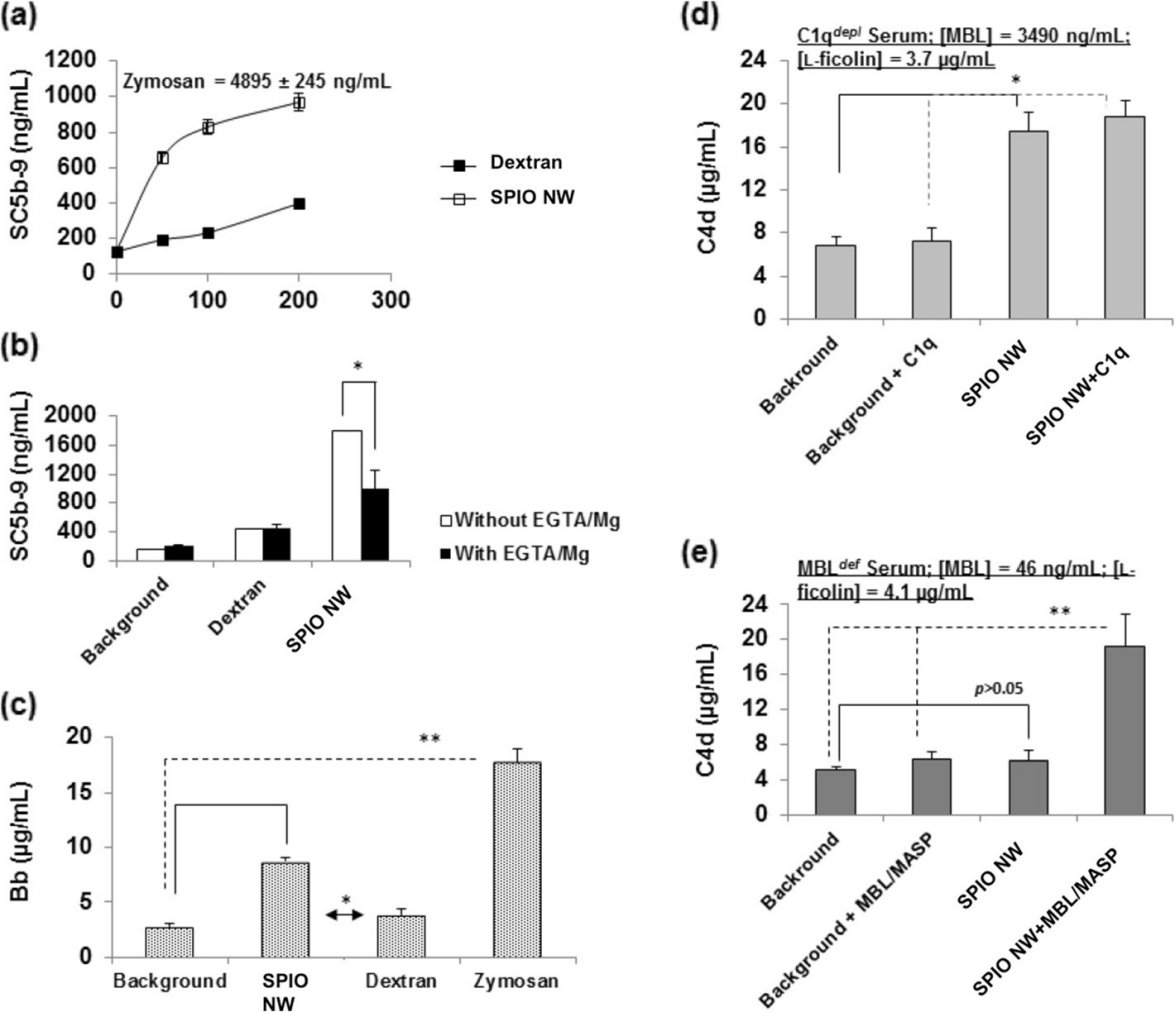
**SPIO-mediated complement activation in defined human sera. (a)** Effect of SPIO NW and dextran concentration on complement activation, based on soluble C5b-9, in a typical human serum from a healthy Caucasian individual. SPIO NW concentration represents an equivalent dextran concentration in the preparation. Zymosan (1 mg/ml) was used as positive control for monitoring complement activation; **(b)** contribution of Ca^2+^-sensitive pathways and the AP turnover to dextran- and SPIO-mediated complement activation in the same serum as panel **(a)**. The background level with EGTA alone is similar to EGTA + Mg^2+^ (data not shown); **(c)** dextran and SPIO NW both enhanced the AP turnover as shown in panel as monitored by Bb generation; **(d)** SPIO-mediated complement generation of C4d is independent of C1q suggesting involvement of the LP; **(e)** MBL is required for SPIO-mediated triggering of the LP. The final concentration of iron in panels **(b-e)** was 200 μg/mL (equivalent dextran concentration). C1q^*depl*^ and MBL^*def*^ represent sera immunochemically depleted from C1q and genetically deficient from MBL, respectively. MBL and L-ficolin concentrations are shown in for C1q^*depl*^ and MBL^*def*^ sera. We used serum from a human donor with high titer of MBL to purify MBL/MASP complexes. The same serum was used to deplete C1q. In experiments where we added MBL/MASP to a serum genetically deficient in MBL, the final equivalent concentration of MBL was 1330 ng/mL (to resemble average MBL concentration in human serum) *p < 0.05, and **p < 0.01.

We examined the effect of SPIO NW concentration on complement activation in a healthy human serum (Figure 2a). The increasing concentration of SPIO NW increased the level of the nonlytic soluble marker of the terminal pathway (TP) of the complement (SC5b-9). This is a sensitive measure of the activation of the whole complement cascade in serum [18]. Notably, the complement activation by SPIO NW was approximately 80% more than by dextran alone at the similar concentration, thereby confirming previous results [37,38]. Activation of the CP and the LP requires divalent cations in the form of Ca^2+^ and Mg^2+^, whereas Mg^2+^ is essential for the operation of the AP [39]. The results in Figure 2b demonstrate that SPIO-mediated complement activation proceeds in serum supplemented with ethylene glycol tetraacetic acid (EGTA)/Mg^2+^ (10.0 mM/2.5 mM), but the generated levels of SC5b-9 are significantly (p <0.05) lower (45% less) compared with normal serum. This suggests that in addition to Ca^2+^-sensitive pathways (CP and/or LP), there is also a direct enhancement of the AP turnover activity. Following addition of SPIO NW to normal sera, there was an elevation of the fluid-phase alternative pathway marker Bb, confirming the role of the AP in SPIO-mediated complement activation (Figure 2c). SPIO-mediated complement activation through calcium-sensitive pathways may also enhance the AP turnover through the amplification loop of the alternative pathway [39] but this was not investigated. In order to establish through which calcium-sensitive pathways SPIO NW can activate complement, we used C1q-depleted (C1q^*depl*^) and genetically deficient MBL (MBL^*def*^) human sera [14]. The immunochemically depleted C1q serum contained physiological levels of the LP initiators (MBL and L-ficolin) [14]. SPIO NW (200 μg/mL) was capable to enhancing C4d (the cleavage product of C4 and an established marker of both CP and LP activation [14,18,39]) release in C1q^*depl*^ serum compared with the background level, and further addition of C1q to serum (180 μg/mL, corresponding to physiological levels [14]) did not elevate serum levels of C4d (Figure 2d). This strongly suggests that SPIO-mediated calcium-sensitive complement activation is exclusively through the LP. Activation of the LP in human sera proceeds following either the binding of serum MBL or L-ficolin to a LP activating surface [14,40] with subsequent activation of MASP-2 and cleavage of C4. There was no increase in the C4d levels following addition of SPIO NW to the MBL^*def*^ serum, whereas addition of purified MBL/MASP-2 complexes resulted in significant (p <0.05) C4d generation. Since the MBL^*def*^ serum contained physiological levels of L-ficolin, this eliminates a role for L-ficolin in SPIO-mediated activation of the LP. On the basis of these experiments, we therefore conclude that SPIO NW activate complement through MBL-mediated LP as well as the direct AP involvement.

**Figure 3:**
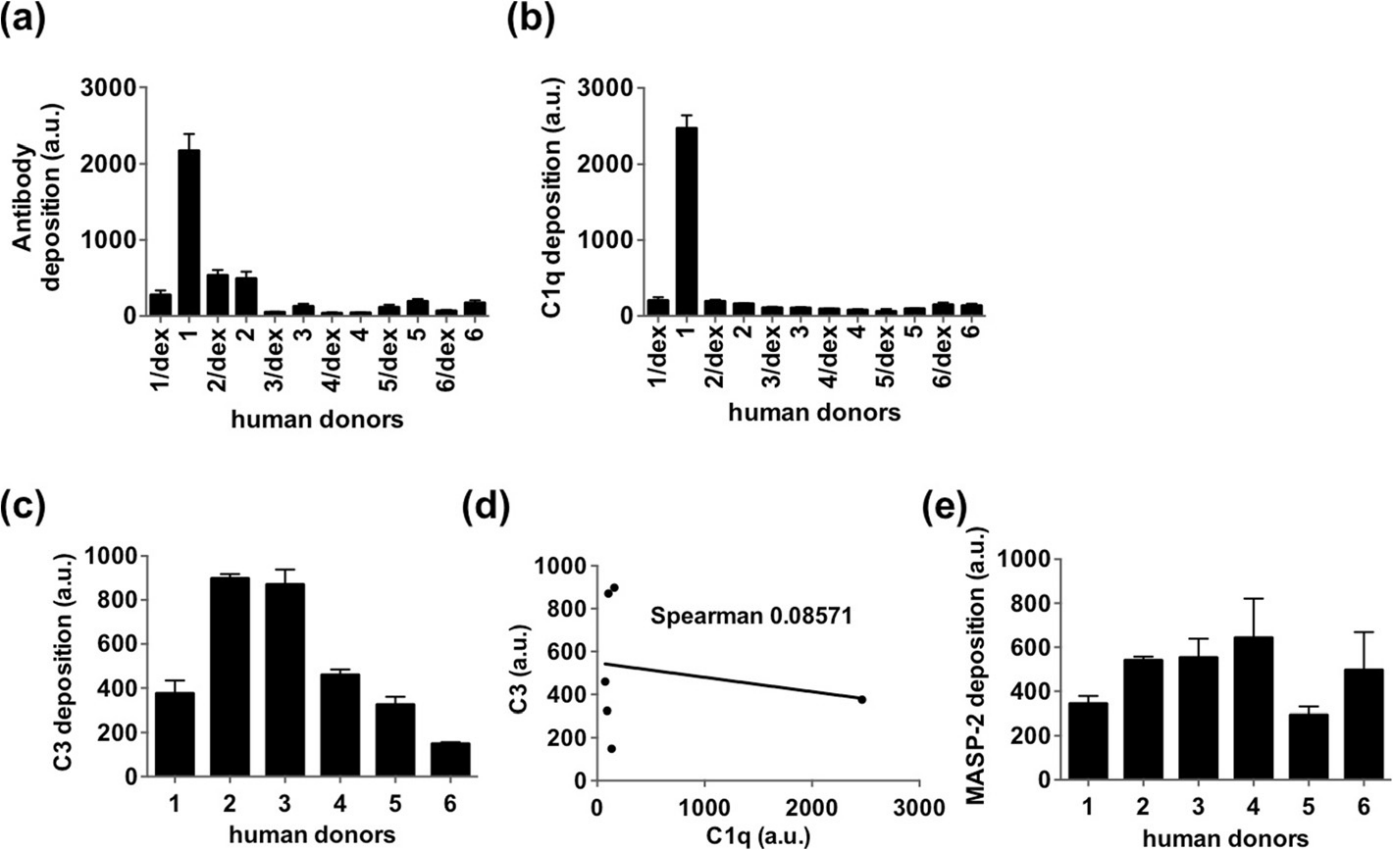
**SPIO NW mediated complement activation in normal human sera from different donors.** Nanoparticles were incubated in human sera as described for mouse experiments, washed and the deposition of proteins was detected with dot blot assay. **(a)** Anti-dextran antibody deposition of SPIO NW in sera of six donors shows that only serum of donor 1 contained a significant amount of anti-dextran antibody as verified by inhibition with free 20 kDa dextran (sample labeled as 1/dex); **(b)** C1q dot blot shows significant dextran-dependent deposition in donor 1; **(c)** C3 deposition shows significant level of complement activation in all six serum samples; **(d)** there was no significant correlation between C1q binding and C3 deposition on SPIO; **(e)** binding of MASP-2 to SPIO NW in human sera. All data represent a mean + SEM of triplicates and the experiments were repeated at least two times. All six samples were from non-smoking, <35 years old Caucasian male subjects.

Despite the fact that SPIO NW did not show any activation of the CP in human sera that we tested so far, several previous studies demonstrated the presence of anti-dextran antibodies in sera of some individuals [13,41] and the involvement of the CP on the surface of nanoparticles due to the presence of anti-dextran IgM antibodies was suggested [13]. We tested sera from six healthy donors for anti-dextran antibodies, C1q deposition and correlation with C3 deposition (Figure 3a). In two out of six samples there was a significant binding of antibodies to SPIO, and only in donor 1 the binding of the antibody was significantly inhibited by free dextran, suggesting specificity (Figure 3b). Also, in the same sample we also observed the binding of C1q that was inhibited by free dextran, suggesting that the CP could be involved in complement activation in sera from that individual (Figure 3c). However, there was no significant correlation (Spearman coefficient 0.08671) between C1q and C3 deposition in the six samples (Figure 3d). At the same time, all serum samples showed similar binding of MASP-2 (Figure 3e) suggesting the binding of the LP components and possible involvement of the LP in all samples. It could be suggested that in sera of individuals who have high titres of antibodies against dextran there is an activation of the CP (in addition to the LP and the AP).

**Figure 4:**
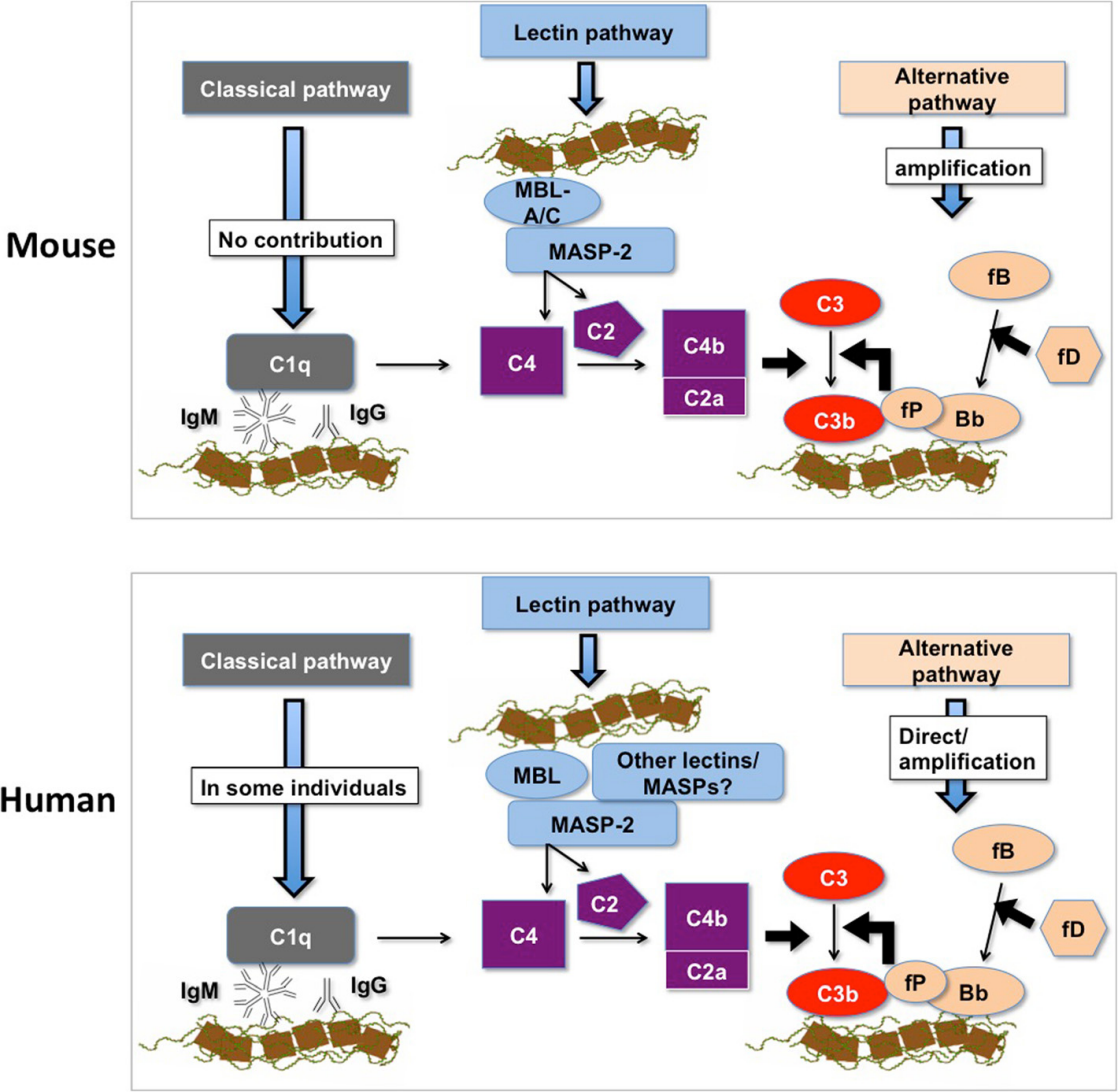
**Summary of the initial steps of complement activation on SPIO NW surface: mouse vs. man.** There is an involvement of the LP in both mouse and human sera. The main difference is the direct involvement of the AP turnover in human sera, and activation of the CP in some human individuals. The role of multiple LP components in humans is not clear at this point.

The initial steps of complement activation and C3b deposition in mouse vs. man are summarized in Figure 4. Our experiments unambiguously demonstrate that SPIO NW trigger complement activation in both mouse and human sera. However, the relative contribution of the pathways is different in mice and humans. In mouse sera, we found that the complement is triggered via the LP, and we did not find evidence for a significant role of the CP. The fact that there was ~50-fold higher C3 deposition in *C1q*^*−/−*^ serum compared to *MBL-A/C*^*−/−*^*C1q*^*−/−*^ serum suggests the predominant contribution of the LP in the complement activation. However, the classical pathway is unstable in mouse sera upon storage and is also highly variable [42], therefore some involvement of the CP in mice cannot be completely ruled out. In humans, the contribution of the CP via anti-dextran antibodies is possible in some individuals, as reflected in Figure 4. The most important difference between mouse and human complement, however, is the direct involvement of the AP turnover independently of the LP in human serum. Direct activation of the AP turnover has been demonstrated for Listeria pathogen [43]. There is also a distant possibility regarding the direct activation of the AP by the LP components, in C2 and C4-dependent and independent fashion [34,35,44], and this aspect will be addressed in a separate study. Lastly, while in mice the experiments in knockout sera indicated the direct involvement of the MBL-A/C and MASP-2, the situation in humans is more complicated. To date, multiple lectins (e.g., MBL, collectins), ficolins and MASPs have been shown to initiate the LP in human sera [30,45,46], and the role of these factors in the complement activation by SPIO NW remains to be elucidated.

Major questions regarding the interaction between nanoparticle surface chemistry and the complement components still remain. Thus, MBLs bind to wide range of sugars, including mannose and glucose [22], and it is likely that lectins also bind to the dextran coat on SPIO, but at this point we did not investigate the exact mechanisms of assembly of the LP components on the nanoparticle surface. One interesting possibility could be that MBLs bind to serum protein corona, rather than through direct binding to the dextran chains. It is also not clear how dextran structure and molecular weight affect the assembly of the complement components, and whether the relative contribution of the pathways depends on the nanoparticle chemistry. Several studies failed to demonstrate a conclusive correlation between molecular weight of dextran and the complement activation by polymeric nanoparticles, with dextran conformation (“loops and trains” vs. “end-on”) postulated as being the most important factor in the activation efficiency [37,38]. The role of nanoparticle surface properties in the complement activation needs to be rigorously addressed in future studies.

In conclusion, our results reveal important similarities and differences between preclinical (mouse) and clinical (human) systems with respect to complement activation by SPIO NW. It is plausible to suggest that the observed immunological behavior of SPIO NW could be applied to Feridex and other variants of dextran SPIO nanoparticles. Although Feridex and other dextran SPIO have been discontinued from the clinical use, the results of our study are critically important for development of new, more safe MRI contrast agents. In that regard, it is clear from our studies that strategies to reduce complement activation by nanoparticles in a mouse system might not necessarily translate into a human system. Mechanistic studies of nanoparticle immune recognition should become an integral part of nanomedicine research and development in order to advance nanomedicine to a new level.

## Methods

### Nanoparticle synthesis and characterization

SPIO NW was prepared by precipitation of Fe^2+^ and Fe^3+^ salts in ammonia in the presence of branched dextran of 20 kDa Mw (Sigma), as described elsewhere in the literature [27,28,47]. Particles were re-suspended in phosphate buffered saline (1xPBS) at 1–2 mg (Fe)/ml and filtered through a 0.2 μm membrane filter. Nanoparticle size (intensity distribution) was measured using a Zetasizer Nano (Malvern Instruments, Worcestershire, UK). For nanoparticle imaging with transmission electron microscopy, the nanoparticle solution in water was placed on Formvar-/carbon-coated grids (Ted Pella, Redding, CA, USA). After 5 min, the grid was gently blotted and air-dried. All the samples were studied without counterstaining. Grids were viewed using a JEOL 1200EX II transmission electron microscope at 75 kV and different instrumental magnifications. Images were captured using a Gatan digital camera.

### Complement activation in mouse sera

In all these studies sera from WT or knock out mice on C57BL/6 J background were used. Mouse sera were collected as described [48] according to the protocols approved by the **IACUC (Institutional Animal Care and Use Committee)** and stored at or below −70°C before use. Each serum sample was subjected less than 2 freeze-thaw cycles, and aliquots were used whenever possible. C3 deposition on SPIO NW was determined by immunoblotting following incubation in normal sera or sera deficient for complement factors and components (obtained from the corresponding mice homozygous for the gene deficiencies [32,33,49-52]). SPIO NW (final concentration 0.4 mg iron/ml in 1× PBS) was incubated with different dilutions of mouse sera (normally 10 μl particles and 30 μl serum) and incubated for 10–30 min at either room temperature of 37°C. Following incubation, the nanoparticles were washed with 1 ml PBS (without calcium or magnesium) four times by ultracentrifugation (Beckman TLA-100 ultracentrifuge, TLA-100.3 rotor, 55.000 rpm for 10 min) and resuspended in PBS at 0.5 mg/ml. As a washing quality control, a separate tube with serum but without nanoparticles was washed using the exact same procedure. Zymosan (2 × 10^9^ particles/mL) was prepared by boiling 4 mg/mL solution of zymosan (Sigma Aldrich) in normal saline for 60 min, washing in PBS twice and resuspending in PBS + 0.1% sodium azide. For dot-blot assay, nanoparticles (1 μl, 0.5 μg iron) were spotted onto a 0.22 μm nitrocellulose membrane (Bio-Rad) in triplicates. The membrane was dried and blocked in 5% dry milk solution in Tween-20/PBS buffer. For western blotting, particles were boiled at 90°C for 5 min in the reducing sample buffer (Bio-Rad), loaded on a Tris-Glycine 4-20% minigel (Life Technologies), the proteins were separated and transferred to a nitrocellulose membrane using iBlot apparatus (Life Technologies). C3 on the membrane was detected with goat anti-mouse C3 polyclonal antibody (MP Biomedicals) and donkey anti-goat 800CW antibody (Li-COR Biosciences, Lincoln, NE). Membranes were scanned at 800 nm with Li-COR Odyssey scanner and the integrated C3 density of each dot was calculated with the NIH ImageJ software. The differences in the C3 deposition between sera were analyzed with non-paired two-sided t-test at 95% CI using Prism software (GraphPad, San Diego).

### Complement activation in human sera

Human sera were either commercially obtained or collected according to the pre-approved IRB (Instutional Review Board) protocol. Details for preparation, characterization and functional assessments of complement pathways as well as determination of MBL and L-ficolin concentrations in normal human serum, C1q-depleted serum and MBL-deficient serum were in accordance with our previous studies [14,39,53]. MBL/MASP-2 preparation and characterization was described previously [54]. To measure complement activation *in vitro*, we determined SPIO- and dextran-induced rise of serum complement activation products C4d, Bb and SC5b-9 using respective ELISA kits (Quidel, San Diego) according to the manufacturer’s protocols as described previously [14,18,39,53]. For measurement of complement activation, the reaction was started by adding the required quantity of SPIO NW (or dextran) to undiluted serum in Eppendorf tubes (either in duplicate or triplicate, depending on experiment) in a shaking water bath at 37°C for 30 min, unless stated otherwise. Reactions were terminated by addition of ‘sample diluent’ provided with assay kits or saline containing 25 mM EDTA. Serum complement activation products were measured following nanoparticle removal by centrifugation. Control incubations contained saline (the same volume as SPIO) for background measurement of complement activation products. In some experiments, SPIO-mediated complement activation was monitored in the presence of EGTA/Mg^2+^ (10.0 mM/2.5 mM) as well as following restoration of serum with deficient complement protein. Zymosan (1 mg/mL) was used as a positive control for complement activation throughout. For quantification of complement activation products, standard curves were constructed using the assigned concentration of each respective standard supplied by manufacturer and validated as described earlier [14,18,39,53]. The efficacy of SPIO NW (or dextran) treatments was established by comparison with baseline levels using paired *t*-test; correlations between two variables were analysed by linear regression, and differences between groups (when necessary) were examined using ANOVA followed by multiple comparison with Student-Newman-Keuls test.

Binding studies of anti-dextran antibody, C1q and C3 in human sera was performed on samples from non-smokers, age <35, males, white. Particles were incubated with sera, washed in PBS with 2 mM Ca^2+^/Mg^2+^ (for C1q) or in Ca^2+^/Mg^2+^ free PBS (for C3 and antibody), and immunoblotted as described above. Anti-dextran antibody was detected using IRDye 800CW (Li-COR) labeled anti-human antibody that is reactive with both IgG and IgM, C1q was probed with goat anti-human/mouse C1q (Santa Cruz Biotechnology) and then detected with donkey anti-goat IRDye 800CW, C3 was probed with goat anti-human C3 (MP Biomedicals) and detected with donkey anti-goat IRDye 800CW. Integrated density of the spots was calculated with ImageJ. For dextran inhibition studies, sera were preincubated with 10 mg/mL of 20 kDa dextran for 5 min prior to addition of nanoparticles. Correlation between C3 and C1q deposition was determined with Spearman two-tailed non-parametric test with 95% confidence interval.

## Additional files

Additional file 1: Figure S1 Physicochemical properties of SPIO nanoworms. Additional file 2: Figure S2 Complement products on the surface of Feridex and SPIO nanoworms.

## References

[1] Figuerola A, Di Corato R, Manna L, Pellegrino T (2010). From iron oxide nanoparticles towards advanced iron-based inorganic materials designed for biomedical applications. Pharmacol Res.

[2] Gupta AK, Gupta M (2005). Synthesis and surface engineering of iron oxide nanoparticles for biomedical applications. Biomaterials.

[3] Bulte JW, Kraitchman DL (2004). Iron oxide MR contrast agents for molecular and cellular imaging. NMR Biomed.

[4] Ricklin D, Hajishengallis G, Yang K, Lambris JD (2010). Complement: a key system for immune surveillance and homeostasis. Nat Immunol.

[5] Janssen BJC, Huizinga EG, Raaijmakers HCA, Roos A, Daha MR, Nilsson-Ekdahl K, Nilsson B, Gros P (2005). Structures of complement component C3 provide insights into the function and evolution of immunity. Nature.

[6] Ali YM, Lynch NJ, Haleem KS, Fujita T, Endo Y, Hansen S, Holmskov U, Takahashi K, Stahl GL, Dudler T, Girija UV, Wallis R, Kadioglu A, Stover CM, Andrew PW, Schwaeble WJ: **The lectin pathway of complement activation is a critical component of the innate immune response to pneumococcal infection.** *Plos Pathog* 2012, **8:**e1002793. 10.1371/journal.ppat.1002793 PMC3390405 22792067

[7] Moghimi SM, Farhangrazi ZS (2013). Nanomedicine and the complement paradigm. Nanomedicine.

[8] Helmy KY, Katschke KJ, Gorgani NN, Kljavin NM, Elliott JM, Diehl L, Scales SJ, Ghilardi N, van Lookeren CM (2006). CRIg: a macrophage complement receptor required for phagocytosis of circulating pathogens. Cell.

[9] Taylor PR, Martinez-Pomares L, Stacey M, Lin HH, Brown GD, Gordon S (2005). Macrophage receptors and immune recognition. Annu Rev Immunol.

[10] Peng Q, Li K, Sacks SH, Zhou W (2009). The role of anaphylatoxins C3a and C5a in regulating innate and adaptive immune responses. Inflamm Allergy Drug Targets.

[11] Szebeni J (2005). Complement activation-related pseudoallergy: a new class of drug-induced acute immune toxicity. Toxicology.

[12] Andersen AJ, Hashemi SH, Andresen TL, Hunter AC, Moghimi SM (2009). Complement: alive and kicking nanomedicines. J Biomed Nanotechnol.

[13] Pedersen MB, Zhou X, Larsen EK, Sorensen US, Kjems J, Nygaard JV, Nyengaard JR, Meyer RL, Boesen T, Vorup-Jensen T (2010). Curvature of synthetic and natural surfaces is an important target feature in classical pathway complement activation. J Immunol.

[14] Andersen AJ, Robinson JT, Dai H, Hunter AC, Andresen TL, Moghimi SM (2013). Single-walled carbon nanotube surface control of complement recognition and activation. ACS Nano.

[15] Peracchia MT, Vauthier C, Passirani C, Couvreur P, Labarre D (1997). Complement consumption by poly(ethylene glycol) in different conformations chemically coupled to poly(isobutyl 2-cyanoacrylate) nanoparticles. Life Sci.

[16] Pham CT, Mitchell LM, Huang JL, Lubniewski CM, Schall OF, Killgore JK, Pan D, Wickline SA, Lanza GM, Hourcade DE (2011). Variable antibody-dependent activation of complement by functionalized phospholipid nanoparticle surfaces. J Biol Chem.

[17] Al-Hanbali O, Rutt KJ, Sarker DK, Hunter AC, Moghimi SM (2006). Concentration dependent structural ordering of poloxamine 908 on polystyrene nanoparticles and their modulatory role on complement consumption. J Nanosci Nanotechnol.

[18] Moghimi SM, Hamad I, Andresen TL, Jorgensen K, Szebeni J (2006). Methylation of the phosphate oxygen moiety of phospholipid-methoxy(polyethylene glycol) conjugate prevents PEGylated liposome-mediated complement activation and anaphylatoxin production. Faseb J.

[19] Salvador-Morales C, Zhang L, Langer R, Farokhzad OC (2009). Immunocompatibility properties of lipid-polymer hybrid nanoparticles with heterogeneous surface functional groups. Biomaterials.

[20] Moore A, Weissleder R, Bogdanov A (1997). Uptake of dextran-coated monocrystalline iron oxides in tumor cells and macrophages. J Magn Reson Imaging.

[21] Simberg D, Park JH, Karmali PP, Zhang WM, Merkulov S, McCrae K, Bhatia SN, Sailor M, Ruoslahti E (2009). Differential proteomics analysis of the surface heterogeneity of dextran iron oxide nanoparticles and the implications for their in vivo clearance. Biomaterials.

[22] Turner MW (1996). Mannose-binding lectin: the pluripotent molecule of the innate immune system. Immunol Today.

[23] Karmali PP, Chao Y, Park JH, Sailor MJ, Ruoslahti E, Esener SC, Simberg D (2012). Different effect of hydrogelation on antifouling and circulation properties of dextran-iron oxide nanoparticles. Mol Pharm.

[24] Chao Y, Karmali PP, Mukthavaram R, Kesari S, Kouznetsova VL, Tsigelny IF, Simberg D (2013). Direct recognition of superparamagnetic nanocrystals by macrophage scavenger receptor SR-AI. ACS Nano.

[25] Chao Y, Makale M, Karmali PP, Sharikov Y, Tsigelny I, Merkulov S, Kesari S, Wrasidlo W, Ruoslahti E, Simberg D (2012). Recognition of dextran-superparamagnetic iron oxide nanoparticle conjugates (Feridex) via macrophage scavenger receptor charged domains. Bioconjug Chem.

[26] Molday RS, MacKenzie D (1982). Immunospecific ferromagnetic iron-dextran reagents for the labeling and magnetic separation of cells. J Immunol Methods.

[27] Jung CW, Jacobs P (1995). Physical and chemical properties of superparamagnetic iron oxide MR contrast agents: ferumoxides, ferumoxtran, ferumoxsil. Magn Reson Imaging.

[28] Jung CW (1995). Surface properties of superparamagnetic iron oxide MR contrast agents: ferumoxides, ferumoxtran, ferumoxsil. Magn Reson Imaging.

[29] Cunnion KM, Hair PS, Buescher ES (2004). Cleavage of complement C3b to iC3b on the surface of Staphylococcus aureus is mediated by serum complement factor I. Infect Immun.

[30] Fujita T, Matsushita M, Endo Y (2004). The lectin-complement pathway–its role in innate immunity and evolution. Immunol Rev.

[31] Takahashi M, Iwaki D, Kanno K, Ishida Y, Xiong J, Matsushita M, Endo Y, Miura S, Ishii N, Sugamura K, Fujita T (2008). Mannose-binding lectin (MBL)-associated serine protease (MASP)-1 contributes to activation of the lectin complement pathway. J Immunol.

[32] Banda NK, Takahashi M, Takahashi K, Stahl GL, Hyatt S, Glogowska M, Wiles TA, Endo Y, Fujita T, Holers VM, Arend WP (2011). Mechanisms of mannose-binding lectin-associated serine proteases-1/3 activation of the alternative pathway of complement. Mol Immunol.

[33] Banda NK, Takahashi M, Levitt B, Glogowska M, Nicholas J, Takahashi K, Stahl GL, Fujita T, Arend WP, Holers VM (2010). Essential role of complement mannose-binding lectin-associated serine proteases-1/3 in the murine collagen antibody-induced model of inflammatory arthritis. J Immunol.

[34] Selander B, Martensson U, Weintraub A, Holmstrom E, Matsushita M, Thiel S, Jensenius JC, Truedsson L, Sjoholm AG (2006). Mannan-binding lectin activates C3 and the alternative complement pathway without involvement of C2. J Clin Invest.

[35] Suankratay C, Zhang XH, Zhang Y, Lint TF, Gewurz H (1998). Requirement for the alternative pathway as well as C4 and C2 in complement-dependent hemolysis via the lectin pathway. J Immunol.

[36] Mestas J, Hughes CCW (2004). Of mice and not men: differences between mouse and human immunology. J Immunol.

[37] Bertholon I, Vauthier C, Labarre D (2006). Complement activation by core-shell poly(isobutylcyanoacrylate)-polysaccharide nanoparticles: influences of surface morphology, length, and type of polysaccharide. Pharm Res.

[38] Labarre D, Vauthier C, Chauvierre C, Petri B, Muller R, Chehimi MM (2005). Interactions of blood proteins with poly(isobutylcyanoacrylate) nanoparticles decorated with a polysaccharidic brush. Biomaterials.

[39] Hamad I, Hunter AC, Moghimi SM (2013). Complement monitoring of Pluronic 127 gel and micelles: Suppression of copolymer-mediated complement activation by elevated serum levels of HDL, LDL, and apolipoproteins AI and B-100. J Control Release.

[40] Roos A, Bouwman LH, Munoz J, Zuiverloon T, Faber-Krol MC, den Houten FC F-v, Klar-Mohamad N, Hack CE, Tilanus MG, Daha MR (2003). Functional characterization of the lectin pathway of complement in human serum. Mol Immunol.

[41] Chacko BK, Appukuttan PS (2003). Dextran-binding human plasma antibody recognizes bacterial and yeast antigens and is inhibited by glucose concentrations reached in diabetic sera. Mol Immunol.

[42] Lachmann PJ (2010). Preparing serum for functional complement assays. J Immunol Methods.

[43] Croize J, Arvieux J, Berche P, Colomb MG (1993). Activation of the human complement alternative pathway by Listeria monocytogenes: evidence for direct binding and proteolysis of the C3 component on bacteria. Infect Immun.

[44] Iwaki D, Kanno K, Takahashi M, Endo Y, Matsushita M, Fujita T (2011). The role of mannose-binding lectin-associated serine protease-3 in activation of the alternative complement pathway. J Immunol.

[45] Hansen S, Selman L, Palaniyar N, Ziegler K, Brandt J, Kliem A, Jonasson M, Skjoedt MO, Nielsen O, Hartshorn K, Jorgensen TJ, Skjodt K, Holmskov U (2010). Collectin 11 (CL-11, CL-K1) is a MASP-1/3-associated plasma collectin with microbial-binding activity. J Immunol.

[46] Liu Y, Endo Y, Iwaki D, Nakata M, Matsushita M, Wada I, Inoue K, Munakata M, Fujita T (2005). Human M-ficolin is a secretory protein that activates the lectin complement pathway. J Immunol.

[47] Park JH, von Maltzahn G, Zhang L, Schwartz MP, Ruoslahti E, Bhatia S, Sailor MJ (2008). Magnetic iron oxide nanoworms for tumor targeting and imaging. Adv Mater.

[48] Banda NK, Hyatt S, Antonioli AH, White JT, Glogowska M, Takahashi K, Merkel TJ, Stahl GL, Mueller-Ortiz S, Wetsel R, Arend WP, Holers VM (2012). Role of C3a receptors, C5a receptors, and complement protein C6 deficiency in collagen antibody-induced arthritis in mice. J Immunol.

[49] Coxon A, Rieu P, Barkalow FJ, Askari S, Sharpe AH, von Andrian UH, Arnaout MA, Mayadas TN (1996). A novel role for the beta 2 integrin CD11b/CD18 in neutrophil apoptosis: a homeostatic mechanism in inflammation. Immunity.

[50] Moller-Kristensen M, Ip WK, Shi L, Gowda LD, Hamblin MR, Thiel S, Jensenius JC, Ezekowitz RA, Takahashi K (2006). Deficiency of mannose-binding lectin greatly increases susceptibility to postburn infection with Pseudomonas aeruginosa. J Immunol.

[51] Botto M (1998). C1q knock-out mice for the study of complement deficiency in autoimmune disease. Exp Clin Immunogenet.

[52] Matsumoto M, Fukuda W, Circolo A, Goellner J, Strauss-Schoenberger J, Wang X, Fujita S, Hidvegi T, Chaplin DD, Colten HR (1997). Abrogation of the alternative complement pathway by targeted deletion of murine factor B. Proc Natl Acad Sci U S A.

[53] Hamada I, Hunter AC, Szebeni J, Moghimi SM (2008). Poly(ethylene glycol)s generate complement activation products in human serum through increased alternative pathway turnover and a MASP-2-dependent process. Mol Immunol.

[54] Tan SM, Chung MC, Kon OL, Thiel S, Lee SH, Lu J (1996). Improvements on the purification of mannan-binding lectin and demonstration of its Ca(2+)-independent association with a C1s-like serine protease. Biochem J.

