# Supplementary figures and images for: Mechanisms of complement activation by dextran-coated superparamagnetic iron oxide (SPIO) nanoworms in mouse versus human serum
Source: Part Fibre Toxicol. 2014 Nov 26;11:64. doi: 10.1186/s12989-014-0064-2 (PMC4247556; doi:10.1186/s12989-014-0064-2)

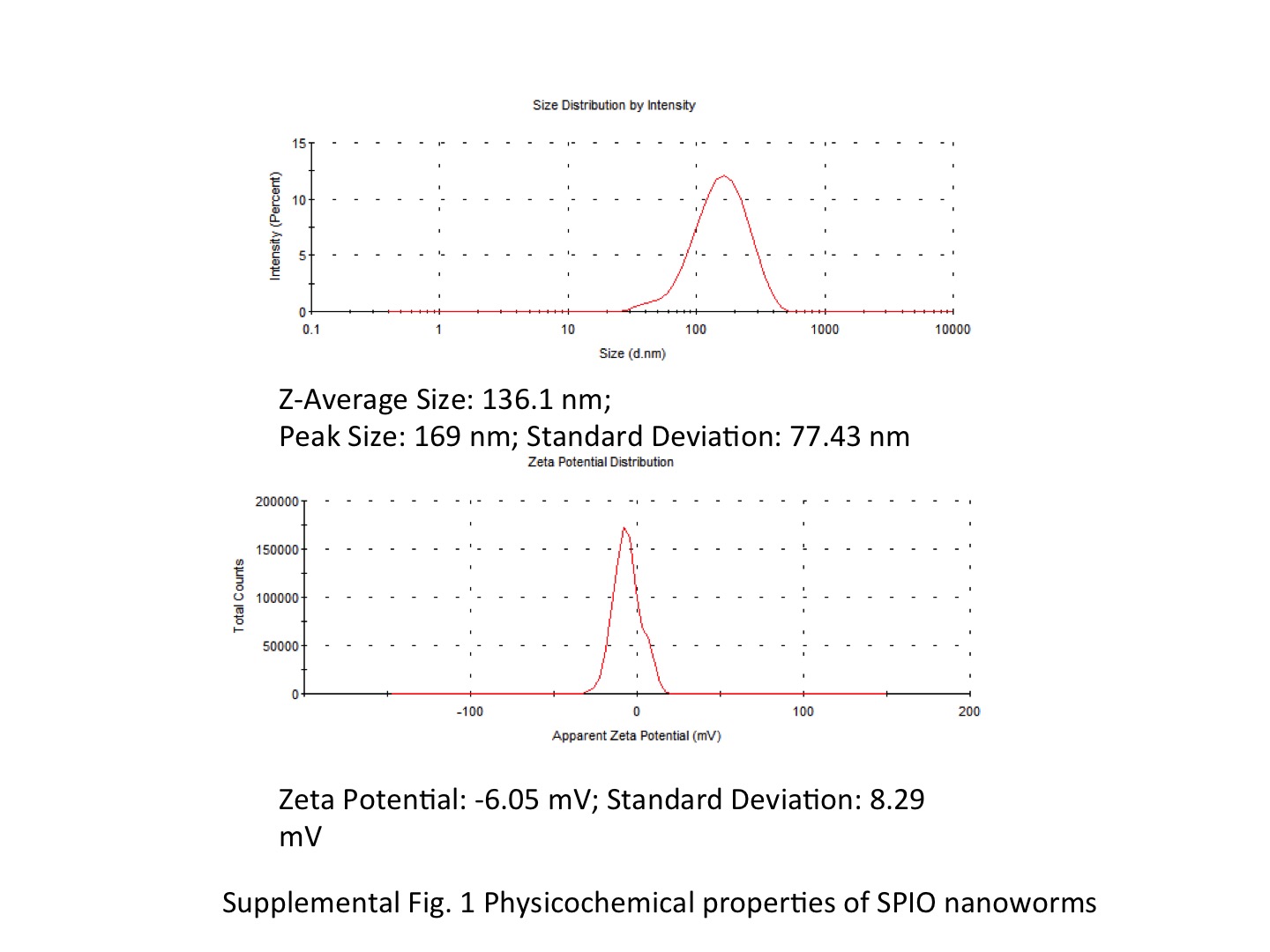

Supplement: Additional file 1: Figure S1 — Physicochemical properties of SPIO nanoworms. [file 12989_2014_64_MOESM1_ESM.jpeg]

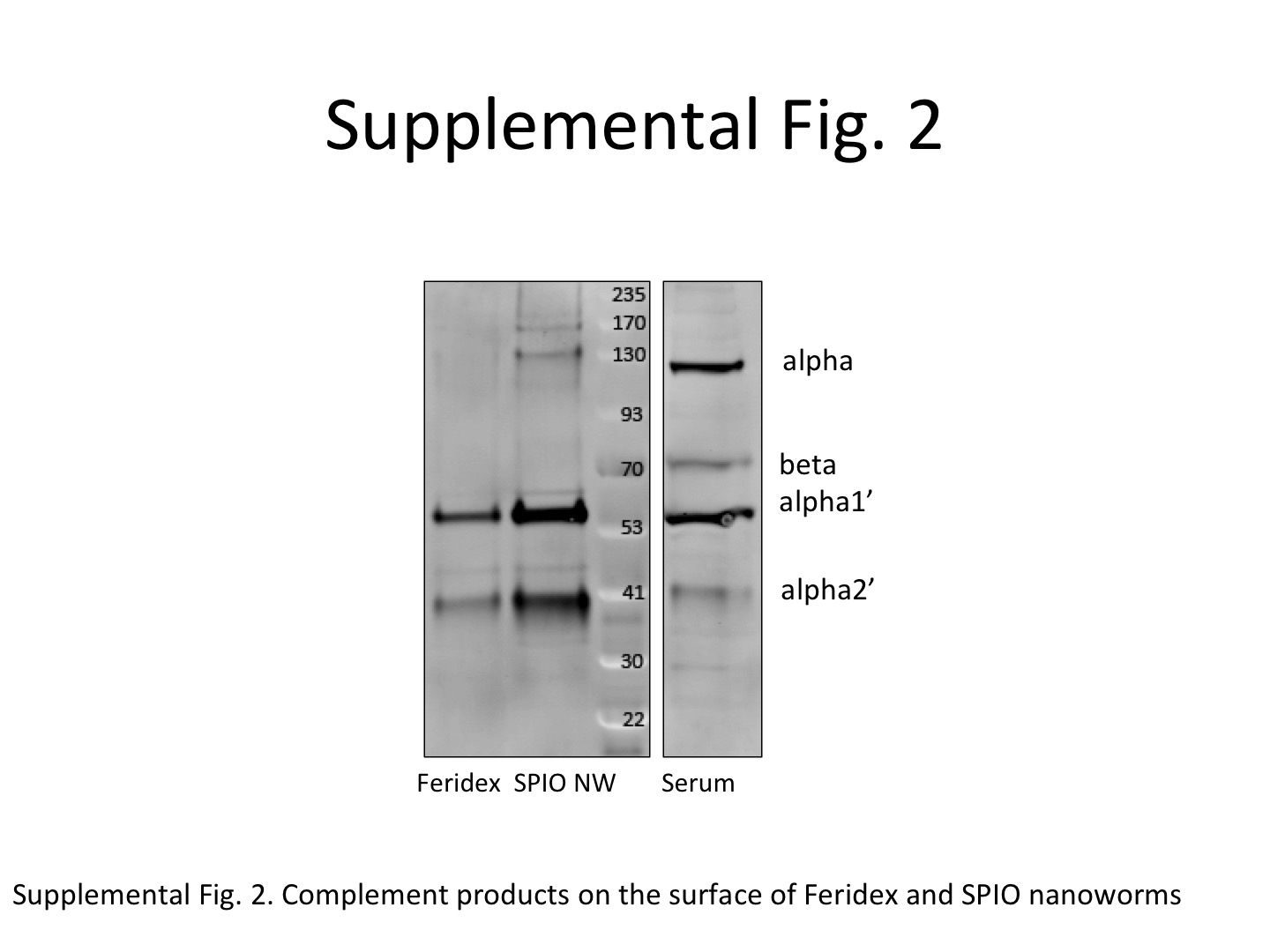

Supplement: Additional file 2: Figure S2 — Complement products on the surface of Feridex and SPIO nanoworms. [file 12989_2014_64_MOESM2_ESM.jpeg]
